# The Clinical and Laboratory Profiles of a Deletional α2-Globin Gene Polyadenylation Signal Sequence (AATAAA > AATA--) [HBA2:c.*93_*94delAA]: The Malaysian Experience

**DOI:** 10.3390/diagnostics15101284

**Published:** 2025-05-20

**Authors:** Norafiza Mohd Yasin, Syahzuwan Hassan, Nur Aisyah Aziz, Faidatul Syazlin Abdul Hamid, Ezalia Esa, Ezzanie Suffya Zulkefli, Rohana Ghazali, Syirah Nazirah Tajuddin, Mohd Nazif Darawi, Yuslina Mat Yusoff, Cornelis L. Harteveld

**Affiliations:** 1 Haematology Unit, Cancer Research Centre (CaRC), Institute for Medical Research (IMR), National Institute for Health (NIH), Shah Alam 40170, Selangor, Malaysia; syah606@gmail.com (S.H.); naisyah.aziz@gmail.com (N.A.A.); syazlinabdulhamid@gmail.com (F.S.A.H.); ezalia@moh.gov.my (E.E.); suffya_zul@hotmail.com (E.S.Z.); yuslina.my@moh.gov.my (Y.M.Y.); 2 Leiden University Medical Centre, 2333 ZA Leiden, The Netherlands; c.l.harteveld@lumc.nl; 3 Haematology Unit, Department of Pathology, Hospital Melaka, Melaka 75400, Malaysia; rohana5326@moh.gov.my; 4 Haematology Unit, Department of Pathology, Hospital Tuanku Jaafar, Seremban 70300, Negeri Sembilan, Malaysia; syirah.nazirah@moh.gov.my; 5 Department of Medical Diagnostics, Faculty of Health Sciences, University Selangor, Shah Alam 40000, Selangor, Malaysia; nazif@unisel.edu.my

**Keywords:** non-deletional α-thalassaemia, polyadenylation mutation, AATA(--AA), Hb H disease

## Abstract

Poly A (AATAAA > AATA--) [HBA2:c.*93_*94delAA] is a rare α-variant reported in our population. It is caused by 2 bp deletion (--AA) in the α2 poly A sequence, leading to a significant α–thalassaemia phenotype. **Background/Objectives**: This study describes the haematological parameters, phenotype, and genotype characteristics of AATA(--AA) in the Malaysian population. **Methods**: The study was carried out on 17 177 cases referred to the Institute for Medical Research, Malaysia, for further diagnosis of α-thalassaemia in a five-year period. Alpha-Gap and ARMS-PCR were performed to detect common α-thalassaemia, followed by *HBA1* and *HBA2* genes sequencing and multiplex ligation-dependent probe amplification (MLPA). Haematological parameters among various groups with the AATA(--AA) allele were presented in this study. **Results**: Thirty-two patients with AATA(--AA) displaying an α–thalassaemia-like phenotype were analysed. They comprised 22 (68.75%) AATA(--AA) carriers, 2 (6.25%) compounds with 3.7 deletion, 2 (6.25%) compounds with --SEA deletion, 1 (3.12%) AATA(--AA) homozygote, and 3 (9.37%) compounds of Hb Adana, Hb CS, and Hb Pakse with co-inheritance Hb E, respectively. Most of the patients with AATA(--AA) compounds with the α-variant exhibited a significant phenotype between moderate to severe thalassaemia, especially cases with compound α^−AA^α/α^Adana^α. **Conclusions**: AATA(--AA) is a significant pathogenic variant that should be diagnosed to prevent significant thalassaemia phenotype or transfusion-dependent thalassaemia.

## 1. Introduction

Alpha-thalassemia is an autosomal recessive disorder characterized by microcytic hypochromic anaemia. It primarily arises from large fragment deletions (copy number variations, or CNVs) or point mutations (referred to as single-nucleotide variations, or SNVs) in the regions that encode the α-globin chains. These genetic changes lead to a varying degree of reduced or absent production of α-globin chains [1]. The clinical phenotype is variable, from almost asymptomatic to lethal haemolytic anaemia. Compound heterozygotes and some homozygotes have a moderate to severe form of α-thalassaemia called Hb H disease. Hb Bart’s hydrops foetalis is a lethal form in which no alpha-globin is synthesized. To date, more than 370 mutations have been reported in the public IthaGenes database. Among these mutations, over 130 are deletions, while more than 220 are non-deletional mutations, with the remaining mutations falling into other categories [2]. Detailed information about these variations is regularly recorded and updated in the IthaNet portal (http://www.ithanet.eu).

Thalassaemia is the most common monogenic disorder globally and prevalent in Mediterranean countries, Southeast Asia (SEA), Africa, the Middle East, and the Indian subcontinent [1,3]. It is estimated that 5% of the world’s population are carriers of a defective α-thalassaemia gene [1]. Alpha thalassemia is particularly common in Southeast Asia. A meta-analysis of α-thalassemia in Southeast Asia found varying frequencies of deletional and non-deletional α-thalassemia variants, with Malaysia having a significant portion of its population affected by these mutations, which accounted for 17.3% [4].

Non-deletional α-thalassaemia are commonly affecting the dominant α2-globin gene (*HBA2*), resulting in a more severe clinical phenotype than those affecting the α1-globin (*HBA1*) gene [5]. Some of the non-deletional α-thalassaemia may produce unstable α-chain variants. In a homozygous state, these variants may produce the Hb H phenotype, and in combination with the α^0^–thalassaemia allele, severe to intermediate hydrops foetalis syndrome [6,7] has been reported. In Malaysia, non-deletional α-thalassemia, such as Cd 142 (TAA > CAA) Hb Constant Spring (Hb CS) (HBA2:c.427T > C) and Cd 59 (GGC > GAC) Hb Adana (HBA2:c.179G > A), is notably prevalent [8,9]. A few rare alpha variants have been reported among the Malaysian population, including Hb Singapore [10] and Hb Ube-2 [11].

The AATA(--AA) (*HBA2*:c.*93_*94delAA) is a rare non-deletional α-thalassaemia and also described as α^TIndia^ [12,13]. It involves a highly conserved sequence (polyadenylation side) of the α2-globin gene. This mutation, predominantly reported among the Indian population, has never been documented in the Malaysian population and may be under-reported. Poly A signal mutations are caused by variable substitutions or deletions on the *HBA2* globin gene [14]. Five polyadenylation signal mutations in the *HBA2* gene have been described. Harteveld et al. [12] reported a novel polyadenylation signal mutation that involved two base deletions, AATAAA > AAT--(α^TIndia^), in the α2-globin gene in 1993; the α^TSaudi^ mutation (AATAAA → AATAAG; *HBA2*: c.*94A > G) was first reported in Saudi Arabia in 1988 [15] and then in Kuwait [16,17], while the AATAAA → AATGAA; *HBA2*: c.*92A > G (α^TTurkish^) mutation was reported in a Turkish family [18,19]. In 2009, Harteveld et al. reported a new polyadenylation site mutation on the α2-globin gene, AATAAA → AATAAC; *HBA2*:c.*94A > C, in Surinamese woman, together with another case of compound (α^TTurkish^/α^TSaudi^) in North African women [19]. Depending on the type of mutation, specific mutations were prevalent in certain ethnic backgrounds. Recently, a novel Poly A (AATAAA > AA-AAA) (HBA2:c.*91delT) mutation was genotyped by third-generation sequencing (TGS) in a patient who presented with severe non-deletional Hb H disease with blood transfusion dependence since infancy [20].

To our knowledge, data on this mutation are limited to a few cases or family reports, with the largest cohort involving 21 patients in India [17]. Over the past 6 years, we have identified and reported 32 cases of AATA(--AA), representing the largest dataset of this mutation in the literature. This study describes the haematological parameters, phenotype, and genotype characteristics of AATA(--AA) in the Malaysian population.

## 2. Materials and Methods

This is a retrospective cross-sectional study of 17,177 samples that were referred to the Institute for Medical Research (IMR) from 2017 to 2023. Data were retrieved from the IMR database. Blood samples were sent to the IMR from various hospitals nationwide for further diagnosis of α-thalassemia after testing negative for common α-thalassemia. The inclusion criteria include individuals with AATA(--AA), identified by Sanger sequencing, in either heterozygous, compound heterozygous, or co-inheritance with other thalassemia variants. In total, 32 cases had the AATA(--AA) mutation. Haematological parameters and clinical phenotypes were retrieved from the medical records.

### 2.1. Mutation Analysis

**Figure 1:**
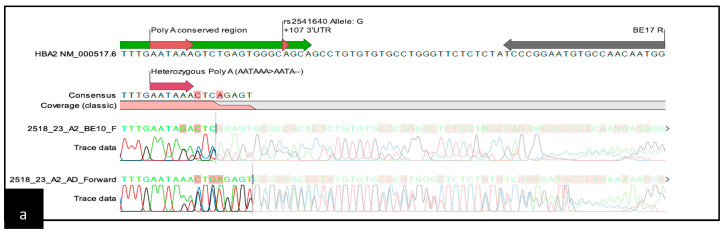

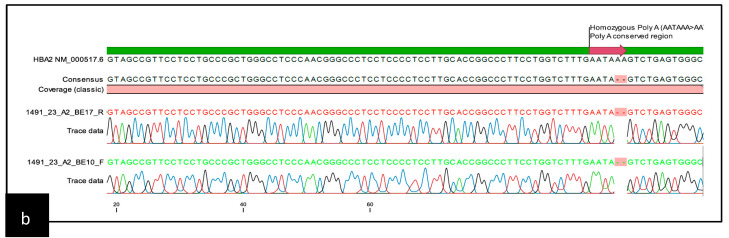
Schematic representation of the sequencing template containing the chromatogram: (**a**) chromatogram pattern for heterozygous AATA(--AA); (**b**) chromatogram pattern for homozygous AATA(--AA).

Common α-thalassemia was ruled out by multiplex GAP and ARMS-PCR [21,22]. For further DNA analysis of the α-thalassemia, whole-blood specimens were collected in K3EDTA tubes. Genomic DNA extraction was performed using the QiaAmp Blood Kit and QIAsymphony DNA Kit (Qiagen Inc., Valencia, CA, USA). The DNA concentration and quality were measured using a NanoDrop 1000 Spectrophotometer (Thermo Fisher Scientific Inc., Wilmington, DE, USA). All DNA samples underwent Sanger sequencing of the *HBA1* and *HBA2* genes. The primer sequences are depicted in Figure S1 (Supplementary Data) including the position and orientation of the primers. Four sequencing reactions were performed for each sample: two for the α1 gene (BE10-F and BE12-R), and two for the α2 gene (BE10-F and BE17-R). Additional reads using an AD Forward primer were added for *HBA2* sequencing. The amplicons were electrophoresed using 1.2% agarose gel and visualized by the Axygen Gel Documentation System (Corning, New York, NY, USA). Following PCR product purification, cycle sequencing was carried out using the BigDye^®^ Terminator v3.1 Cycle Sequencing Kit. Sequencing was performed with the ABI 3730XL DNA Analyzer (Applied Biosystems, Foster City, CA, USA). Sequencing data were analysed using CLC Main Workbench Version 6.9 (Qiagen© GmbH, Hilden, Germany). Examples of the heterozygous and homozygous AATA(--AA) are shown in Figure 1.

### 2.2. Statistical Analysis

Demographic data, including age, state, and ethnicity of AATA(--AA), were analysed by descriptive analysis. Means were reported with standard deviation (SD) and medians with interquartile range (IQR). Data and results were presented in the form of figures and tables.

### 2.3. Ethics Approval

This study was conducted according to the Declaration of Helsinki and approved by the Medical Research and Ethics Committee (MREC) (NIH.800-4/4/1Jld.146(22), the Ministry of Health, and the regional ethical board in Malaysia. Informed consent for clinical information and molecular genotyping was obtained before blood sampling.

## 3. Results

### 3.1. Demographic Profile

Thirty-two patients were studied, comprising seven males (21.8%) and fifteen females (46.8%). The patients’ ages ranged from 2 to 56 years, with a median age of 26 years. Most of the cases were of Malay ethnicity (90.6%), followed by Chinese (6.25%) and Siamese (3.13%). All patients were from West Malaysia.

### 3.2. Genotyping Group

The patients were divided into seven genotype groups as shown in Table 1. The heterozygous AATA(--AA) had the highest incidence (*n* = 22/32, 68.75%). The compound heterozygous AATA(--AA) and non-deletional α-thalassaemia comprised compound heterozygous with Cd 142 (TAA > CAA) Hb Constant Spring (HBA2:c.427T > C), Cd 59 (GGC > GAC) Hb Adana (HBA2:c.179G > A), and Cd 142 (TAA > TAT) Hb Pakse (HBA1:c.264C > G). The compound heterozygous Hb Pakse/AATA(--AA) had co-inheritance with Codon 26 (GAG > AAG) Hb E (HBB:c.79G > A). Other genotype groups involved single cases of compound heterozygous AATA(--AA)/-α^3.7^ and AATA(--AA)/--^SEA^ with homozygous AATA(--AA).

### 3.3. Haematological Characteristics and Clinical Phenotype

A heterozygous carrier of AATA(--AA) presented with an α–thalassaemia phenotype based on the levels of MCV (mean of 69.8 ± 3.07), MCH (22.2 ± 1.57), and RDW (15.38 ± 1.08). They were clinically asymptomatic, with a mean Hb of 12.4 g/dL ± 1.42. The Hb typing was normal, with no additional peak being detected.

**Figure 2:**
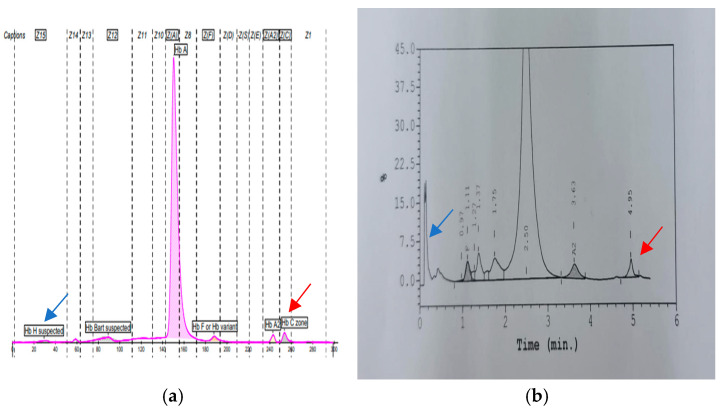
(**a**,**b**): A representative result of compound heterozygous AATA(--AA) with Hb Constant Spring. The blue arrow represents the Hb H peak in CE (0.6%) and HPLC (pre-run peak). The red arrow represents the Hb CS peak with CE (2.1%) and (1.5%) in HPLC. Low HbA_2_ levels were seen, with percentage values of 2.1% and 1.5% in CE and HPLC, respectively.

The homozygous AATA(--AA) patient was a 37-year-old woman who had been misdiagnosed as thalassaemia minor since childhood. However, at the age of 23 years, she presented with severe anaemia, requiring regular blood transfusions. She had hepatosplenomegaly of 3 cm and 5 cm, respectively. Her ferritin level was 2868 ug/dL with a pre-transfusion Hb level between 6.5 and 7.5 g/dL. No documentation on endocrine complications was observed. Her Hb typing showed features of Hb H disease with fraction eluting at a pre-run peak in HPLC, an Hb H peak of 23.1% in the CE, and a low HbA_2_ level (1.6%). Her peripheral blood film showed hypochromic microcytosis with marked anisopoikilocytosis and reticulocytosis of 5.1%. An initial DNA analysis was negative for common deletional and non-deletional α-thalassaemia. Direct sequencing of the HBA2 gene showed homozygous AATA(--AA) mutations (Figure 2b). Possible deletional α-thalassaemia was ruled out by α-MLPA (MRC-Holland, Amsterdam, The Netherland). She was treated as transfusion-dependent thalassaemia with six weekly transfusions and required iron chelation therapy.

A more severe phenotype was observed in the AATA(--AA)/Hb Adana (α^CD59G>A^α/α^--AA^α) patient. She was screened early due to similar health issues in her sibling, who was misdiagnosed as β-thalassemia, and both died at a young age. At 1.5 months old, she presented with hepatosplenomegaly and severe anaemia, with a haemoglobin level of 5.6 g/dL. Initially, she was diagnosed as having β-thalassemia in view of her severe phenotype at a young age, and unfortunately, no documentation was available regarding her condition at birth. The patient required blood transfusions every four weeks and underwent splenectomy at the early age of 9 years old. Her MRI T2* scan showed severe iron overload. Her ferritin level was 7271 μg/L. She was placed on combined iron chelation therapy with deferoxamine and deferiprone. Additionally, she developed secondary ovarian failure and received hormone replacement therapy.

As expected, we observed a milder phenotype in the compound heterozygous AATA(--AA) with Hb CS (α-^AA^α/α^CS^α) cases compared to the AATA(--AA)/Hb Adana. The mean Hb, MCV, MCH, and RDW were 8.9 g/dL ± 0.61, 66.0 fL ± 2.75, 18.8 pg ± 0.20, and 22.3% ± 3.34%, respectively. All three cases presented with a mild to moderate intermediate phenotype. Interestingly, their CS peaks were elevated to more than 1% (ranges 1.3–2%), with a significantly low HbA2 level (ranges 1.3–1.9%) (Figure 2). No documented hepatosplenomegaly was recorded in any of these cases. One of them required transfusion during episodes of acute gastroenteritis.

The compound α^−AA^α/^--SEA^ showed variable phenotypes. The first patient was presented at the age of 2 years and 11 months old with Hb of 9.2 g/dL. He was accidentally noted to be pallor, with enlargement of the liver and spleen, 4 cm and 6 cm below the subcostal region, respectively. The full blood picture (FBP) showed evidence of haemolysis with hypochromic microcytic, anisopoikilocytosis, numerous target cells, pencil cells, and nucleated RBCs being seen. The Hb typing showed a low HbA2 level of 1.9% with zone 15 (HbH of 18.5%). No thalassaemic facies was reported; however, he received blood transfusions during fever and infection. No endocrine complications were reported. He was growing well and started a Folic acid supplement. On average, he received 2–3 blood transfusions a year, and the baseline Hb level was 8.5 to 9.4 g/dL. Initially, he was on iron chelation therapy (Deferasirox 500 mg daily) due to high serum ferritin levels but stopped after one year with serum ferritin of 323 ug/dL. The latest MRI T2* showed mild iron loading. Conversely, the other patient presented earlier at the age of 6 months and was started on regular transfusion (3 weekly) since diagnosis, with a pre-transfusion Hb level between 9 and 10 g/dL. He had huge splenomegaly, and the disease was complicated with pancytopenia that was secondary to hypersplenism. MRI T2* showed moderate to severe iron overload with a recent ferritin level of 3887 μg/L, and the patient was on combined deferoxamine and deferiprone iron chelation therapy.

Other genotypes presented with mild phenotypes and unremarkable physical examinations. The complete blood counts and Hb typings are summarized in Table 1.

## 4. Discussion

The AATA(--AA) mutation was first described in an Indian family presenting with persistent hypochromic microcytosis and an α–thalassemia-like phenotype based on the α/β globin chain ratio [12]. The variant has been reported among Thai, Asian, and Indian populations [12,23,24,25]. The α/β globin chain ratio is a direct measure of the severity of α-thalassemia. A ratio below 0.8 indicates α-thalassemia, with specific values corresponding to different numbers of deleted α genes. A ratio of 0.75 is associated with the loss of one α gene (-α/αα), 0.5 with two α genes (--/αα), and 0.25 with three α genes (--/-α) [1]. Studies have reported a range of clinical phenotypes associated with the α2 Poly A AATA(--AA) mutation. A moderately severe α-thalassemia phenotype was observed in an Australian family carrying this mutation in combination with -α^3.7^ deletion [26], while a British family of Pakistani origin with a homozygous AATA(--AA) mutation exhibited a severe phenotype of Hb H hydrops foetalis syndrome [27]. This mutation, in combination with other α-thalassemia mutations, can lead to severe phenotypes, such as Hb H disease and hydrops fetalis syndrome [24,27]. The two-nucleotide deletion in the polyadenylation sequence AATA(--AA) disrupts not only the expression of the α2 gene but also affects the expression of the α1 gene in cis due to transcriptional interference [12,13,19].

Although the incidence of this mutation in many populations is not well defined, it is likely underestimated, as it is often not included in routine α-thalassemia genotyping panels. In our population, Hb H disease due to the interaction between Poly A mutations with other α-thalassaemia is rare. Here, we report severe phenotypes in cases with homozygous AATA(--AA), compound heterozygous with SEA deletion, and compound heterozygous with Hb Adana. Contrary to previous reports, our case of homozygous Poly A AATA(--AA) presented with moderate to severe thalassaemia intermedia rather than Hb H hydrops fetalis syndrome [27]. A similar case was described in an Iranian patient with homozygous AATA(--AA), who was diagnosed with transfusion-dependent Hb H disease [13]. A different poly A defect may have different consequences on the phenotypic expression, as observed in 17 Kuwaiti patients who have mild phenotypes with homozygous presentation for the α2 (AATAAA > AATAAG) poly a mutation [16]. This could indicate that different poly A defects may have different impacts on the phenotypic expression.

All compound heterozygous cases reported in this study with either α^0^ or unstable alpha variants presented later in life, as typically seen in Hb H disease. However, these individuals eventually developed more severe forms of Hb H disease, with some progressing to transfusion-dependent thalassaemia. The presence of these mutations in trans with other unstable haemoglobins, such as Hb Adana, appeared to exacerbate the clinical phenotype. None of the severe phenotypes presented very early except for compound AATA(--AA) with Hb Adana. Compound heterozygous AATA(--AA) with SEA deletion resulted in moderate anaemia, with numerous Hb H inclusion bodies. Despite having a similar genotype, their clinical presentations were different. The first patient became symptomatic at 2 years, whereas the other was symptomatic at 6 months of age and required frequent transfusions. This aligns with a Thai study reporting a severe phenotype associated with this genotype [24].

The Hb H disease caused by the interaction with AATA(--AA) exhibited variable transfusion dependencies and severities. This interaction showed a more severe clinical pattern than what is usually observed in people with the deletion of three α-genes [12]. In contrast, Hb H disease caused by Poly A mutations involving base substitutions, such as the T^Saudi^ or T^Turkish^ mutations, had a milder phenotype without requiring transfusions or leading to serious complications [15,18]. These findings suggest that the AATA(--AA) is associated with a more severe disease course. Heterozygous carriers of the AATA(--AA) mutation exhibited an α–thalassemia phenotype with a mean MCV of 69.8 ± 3.07 fl and MCH of 22.2 ± 1.57 pg, which are consistent with findings reported by Harteveld [12] of the range of values of MCV being 71–74 fl and those for MCH being 24.0–23.6 pg. Conversely, several carriers in the Indian population presented with a milder phenotype characterized by mild hypochromic microcytic anaemia [17].

Previous studies have shown that the -α^3.7^ deletion is the most prevalent α-thalassemia mutation, particularly in tropical and subtropical regions such as the Mediterranean, Southeast Asia, Africa, the Middle East, and the Indian subcontinent [8,28,29]. While α–thalassemia is less common, the --^SEA^ and --^MED^ deletions are prevalent in Southeast Asia and the Mediterranean region, respectively. In Malaysia, besides --^SEA^, the --^THAI^, --^FIL^, and --^GB^ deletions are also common [8,30]. For non-deletional α-thalassemia, Hb Constant Spring is the most common variant in Southeast Asia [14]. While no studies have reported its incidence in the Malaysian population, it is likely underdiagnosed, as this mutation is often not included in the routine α-thalassemia genotyping panels.

Data from our study indicate that this mutation has a significant impact on the thalassemia phenotype and should therefore be included in the routine screening panel for common alpha-thalassemia mutations, not only in our country but also in other endemic regions. The incidence reported in our study may under-represent the true frequency in the population, as the mutation could be missed in the current diagnostic workflow. A study conducted in the UK found that the AATA(--AA) mutation was the second most common non-deletional α-thalassemia [27]. Interestingly, among the Indian population, it has been postulated that this mutation could be the most common cause of non-deletional α-thalassaemia [17].

The polyadenylation signal sequence (AATAAA) is essential for the final mRNA processing by designating the site for the poly(A) tail addition. This tail ensures mRNA stability, promotes efficient export from the nucleus, and facilitates proper translation [31]. When the AATA(--AA) mutation occurs, it hinders the ability of CPSF (Cleavage and Polyadenylation Specificity Factor) to recognize the altered signal, preventing the effective recruitment of other necessary components of the cleavage and polyadenylation machinery. Consequently, the affected mRNA may not be cleaved or properly polyadenylated, causing it to degrade prematurely [32]. This leads to a reduced supply of α2-globin chains, creating an imbalance with β-globin chains, which aggregate into toxic tetramers like Hb H, impairing red blood cell function and resulting in microcytic and hypochromic anaemia. Additionally, this mutation can affect the expression of the α1-globin gene by interfering with transcription along the same DNA strand (cis interference), exacerbating the overall α-globin deficiency [33]. Notably, although the mutation primarily affects the *HBA2* gene, it appears to influence the *HBA1* gene expression as well, resulting in a phenotype resembling α^0^-thalassemia.

This study indicates that even identical genotypes may have a different phenotype, and other complex factors are involved in explaining the genotype–phenotype correlation better. In the future, incorporating bioinformatics approaches to study the AATA(--AA) mutation could offer deeper insights into how the disrupted CPSF binding affects the mRNA stability and polyadenylation efficiency. In silico analyses would complement experimental findings by predicting the mutation’s molecular impact, aiding in the development of better diagnostic tools and enhancing the understanding of its role in severe clinical phenotypes.

### Limitations

This study had several limitations. The severity of the phenotype could have been better demonstrated through mRNA quantitative analysis. However, as this was a retrospective study, fresh samples that were suitable for mRNA expression analysis were not available. In addition, the red cell parameters, particularly the MCV, MCH, and RDW, may have been influenced by co-inheritance with iron deficiency anaemia, which was not thoroughly investigated in this study.

## 5. Conclusions

The incidence of potentially severe AATA(--AA) mutations in our population may be under-reported, as this variant is not included in common genotype panels. Based on our local data (unpublished observations), this AATA(--AA) mutation is the second most significant alpha variant after Hb Adana in our population. This variant warrants further attention and screening due to its significant impact, alongside Hb Constant Spring. The variability in clinical presentation highlights the importance of genetic characterization in managing the disease effectively, not only for β-thalassaemia, Hb E, but also for α^0^ deletions and severe α+ mutations that may cause severe Hb H disease.

## Figures and Tables

**Table 1:** Haematological parameters and haemoglobin (Hb) subtype profiles of AATA(--AA).

Genotype	α^−AA^α/αα	α^−AA^α/α^−AA^α	α^−AA^α/−α^3.7^	α^−AA^α/^--SEA^	α^−AA^α/α^CS^α	α^−AA^α/α^Adana^α	α^−AA^α/α^Pakse^α, β^E^β
*n* (%)	22	1	2	2	3	1	1
RBC (10^6^/uL)	5.5 ± 0.52	4.0	5.9	3.8	4.7 ± 0.29	3.1	4.8
Hb (g/dL)	12.4 ± 1.42	8.0	11.1 ± 1.90	8.3 ± 1.27	8.9 ± 0.61	5.6	8.6
MCV (fL)	69.8 ± 3.07	70.0	58.4 ± 4.94	75.3 ± 2.19	66.0 ± 2.75	91.0	59.3
MCH (pg)	22.2 ± 1.57	20.0	18.5 ± 0.98	22.1 ± 2.96	18.8 ± 0.20	28.0	18.0
RDW-CV (%)	15.38 ± 1.08	38.5	18.4 ± 0.14	21.3	22.3 ± 3.34	27.7	19.4
CE (%)							
Hb A	96.5 ± 3.37	75.8	97.7	79.6	96.0 ± 2.36		82.1
Hb A2	2.4 ± 0.30	1.1	2.3	1.9	1.5 ± 0.3		2.3
Hb F	0.3 ± 0.00						1.1
Hb variant	NA	Z15 (23.1%)	NA	Z15 (18.5%)	** CS (2.1), Z12 (2.7) and Z15 (0.6)		E, 14.5
HPLC (%)							
Hb A	86.0 ± 0.9		87.0		87.2 ± 1.77		
Hb A2	2.6 ± 0.10	1.6	2.3	1.8	2.3 ± 0.10	2.5	
Hb F	0.6 ± 0.72	0.0	1.8	0.5	0.8 ± 0.78	0.5	
Hb Variant	NA	Pre-run peak		Pre-run peak	Pre-run peak, CS (1.5)	Pre-run peak	
Gel electrophoresis pH8.5	NA	Fast band at H region		Fast band at H region		No H band	
H-Inclusion	NA	Positive		Many inclusions seen		Negative	

Abbreviations/Remarks: All the data are presented as mean ± 2SD; ** case presented at 7 years old with features of non-deletional Hb H disease based on CE. RBCs, red blood cells; Hb, haemoglobin; MCV, mean cell volume; MCH, mean cell haemoglobin; MCHC, mean cell haemoglobin concentration; RDW, red cell distribution width; CE, capillary electrophoresis; HPLC, high-performance liquid chromatography, NA; not applicable; α^−AA^α = AATA(--AA)(NG_000006.1:g.34556_34557del), α^CS^α = CD 142 (TAA > CAA) Hb Constant Spring (NG_000006.1:g.34461T > C); α^Adana^α = CD 59 (GGC > GAC) (NG_000006.1: g.34071G > A); α^Pakse^α = CD 142 (TAA > TAT) (NG_000006.1: g.34463A > T); --^SEA^ = SEA deletion (NG_000006.1: g.26264_45564del); −α^3.7^ = 3.7 deletion (NG_000006.1: g.34164_37967de). NA = not applicable.

## Data Availability

The data presented in this study are available upon request from the corresponding author.

## Supplementary Materials

The following supporting information can be downloaded at: https://www.mdpi.com/article/10.3390/diagnostics15101284/s1, Figure S1 including the position, orientation, and sequence details of the primers.

## References

[1] Harteveld C.L., Higgs D.R. (2010). Alpha-thalassaemia. Orphanet. J. Rare Dis..

[2] Kountouris P., Kousiappa I., Papasavva T., Christopoulos G., Pavlou E., Petrou M., Feleki X., Karitzie E., Phylactides M., Fanis P. (2016). The molecular spectrum and distribution of haemoglobinopathies in Cyprus: A 20-year retrospective study. Sci. Rep..

[3] Amid A., Lal A., Coates T.D., Fucharoen S. (2023). Guidelines for the Management of α-Thalassaemia.

[4] Goh L.P.W., Chong E.T.J., Lee P.C. (2020). Prevalence of Alpha(α)-Thalassemia in Southeast Asia (2010–2020): A Meta-Analysis Involving 83,674 Subjects. Int. J. Env. Res. Public Health.

[5] Farashi S., Harteveld C.L. (2018). Molecular basis of α-thalassemia. Blood Cells Mol. Dis..

[6] Nainggolan I.M., Harahap A., Setianingsih I. (2010). Hydrops fetalis associated with homozygosity for Hb Adana [α59(E8)Gly→Asp (α2)]. Hemoglobin.

[7] Lee T., Lai M., Ismail P., Ramachandran V., Tan J., Teh L., Othman R., Hussein N., George E. (2016). Analysis of α1 and α2 globin genes among patients with hemoglobin Adana in Malaysia. Genet. Mol. Res..

[8] Ahmad R., Saleem M., Aloysious N.S., Yelumalai P., Mohamed N., Hassan S. (2013). Distribution of alpha thalassaemia gene variants in diverse ethnic populations in malaysia: Data from the institute for medical research. Int. J. Mol. Sci..

[9] Idris F., Liew C.Y., Seman Z., Mahmud N. (2020). Optimal Mean Corpuscular Haemoglobin (MCH) Cut-Off Value for Differentiating Alpha Plus and Alpha Zero Thalassaemia in Thalassaemia Screening. Mal. J. Med. Health Sci..

[10] Yasin N.M., Musa N.H., Hamid F.S., Hassan S., Aziz N.A., Sahid E.N., Yusoff Y.M., Jaafar A.A., Esa E. (2022). Haematological and Molecular Characteristics of Hb Singapore [HBA2:c. 425G > C] Unique Among the Malays from Kelantan, Malaysia. Malays. J. Human. Genet..

[11] Yasin N.M. (2023). A Rare Interactions Between Heterozygous --SEA Deletion and Hb Ube-2 [α68 (E17) Asn →Asp]; First Reported Case from Malaysia. Biomed. J. Sci. Tech. Res..

[12] Harteveld C.L., Losekoot M., Haak H., Heister G.A., Giordano P.C., Bernini L.F. (1994). A novel polyadenylation signal mutation in the alpha 2-globin gene causing alpha thalassaemia. Br. J. Haematol..

[13] Farashi S., Garous N.F., Ashki M., Vakili S., Zeinali F., Imanian H., Azarkeivan A., Giordano P.C., Najmabadi H. (2015). Homozygosity for the AATAAA > AATA- - Polyadenylation Site Mutation on the α2-Globin Gene Causing Transfusion-Dependent Hb H Disease in an Iranian Patient: A Case Report. Hemoglobin.

[14] Kalle Kwaifa I., Lai M.I., Md Noor S. (2020). Non-deletional alpha thalassaemia: A review. Orphanet J. Rare Dis..

[15] Thein S.L., Wallace R.B., Pressley L., Clegg J.B., Weatherall D.J., Higgs D.R. (1988). The polyadenylation site mutation in the alpha-globin gene cluster. Blood.

[16] Haider M., Adekile A. (2005). Alpha-2-globin gene polyadenylation (AATAAA-->AATAAG) mutation in hemoglobin H disease among Kuwaitis. Med. Princ. Pract..

[17] Deshpande P., Kamalanathan N., Sampath E., George B., Shaji R.V., Edison E.S. (2015). Characterization of Clinical and Laboratory Profiles of the Deletional α2-Globin Gene Polyadenylation Signal Sequence (AATAAA > AATA- -) in an Indian Population. Hemoglobin.

[18] Yüregir G.T., Aksoy K., Cürük M.A., Dikmen N., Fei Y.J., Baysal E., Huisman T.H. (1992). Hb H disease in a Turkish family resulting from the interaction of a deletional alpha-thalassaemia-1 and a newly discovered poly A mutation. Br. J. Haematol..

[19] Harteveld C.L., Oosterhuis W.P., Schoenmakers C.H., Ananta H., Kos S., Bakker Verweij M., van Delft P., Arkesteijn S.G., Phylipsen M., Giordano P.C. (2010). Alpha-thalassaemia masked by beta gene defects and a new polyadenylation site mutation on the alpha2-globin gene. Eur. J. Haematol..

[20] Ren Z.M., Li W.J., Xing Z.H., Fu X.Y., Zhang J.Y., Chen Y.S., Li D.F. (2023). Detecting rare thalassemia in children with anemia using third-generation sequencing. Hematology.

[21] Chong S.S., Boehm C.D., Higgs D.R., Cutting G.R. (2000). Single-tube multiplex-PCR screen for common deletional determinants of α-thalassemia. Blood.

[22] Eng B., Patterson M., Walker L., Chui D.H., Waye J.S. (2001). Detection of severe nondeletional alpha-thalassemia mutations using a single-tube multiplex ARMS assay. Genet. Test..

[23] Hall G.W., Higgs D.R., Murphy P., Villegas A., de Miguel A. (1994). A mutation in the polyadenylation signal of the alpha 2 globin gene (AATAAA-->AATA--) as a cause of alpha thalassaemia in Asian indians. Br. J. Haematol..

[24] Laosombat V., Fucharoen S., Wiriyasateinkul A. (2001). Interaction of the α2 polyadenylation signal mutation (AATAAA → AATA– –) and α^0^-THALASSEMIA (– –^SEA^), resulting in Hb H disease in a Thai patient. Hemoglobin.

[25] Kountouris P., Lederer C.W., Fanis P., Feleki X., Old J., Kleanthous M. (2014). IthaGenes: An Interactive Database for Haemoglobin Variants and Epidemiology. PLoS ONE.

[26] Prior J.F., Lim E., Lingam N., Raven J.L., Finlayson J. (2007). A Moderately Severe α-Thalassemia Condition Resulting From a Combination of the α2 Polyadenylation Signal (AATA*AA*→AATA– –) Mutation and a 3.7 Kb α Gene Deletion in an Australian Family. Hemoglobin.

[27] Henderson S., Chapple M., Rugless M., Fisher C., Kinsey S., Old J. (2006). Haemoglobin H hydrops fetalis syndrome associated with homozygosity for the alpha2-globin gene polyadenylation signal mutation AATAAA-->AATA- -. Br. J. Haematol..

[28] Azma R.Z., Ainoon O., Hafiza A., Azlin I., Noor Farisah A.R., Nor Hidayati S., Noor Hamidah H. (2014). Molecular characteristic of alpha thalassaemia among patients diagnosed in UKM Medical Centre. Malays. J. Pathol..

[29] Rahimah A.N., Nisha S., Safiah B., Roshida H., Punithawathy Y., Nurul H., Syahzuwan H., Zubaidah Z. (2012). Distribution of alpha thalassaemia in 16 year old Malaysian Students in Penang, Melaka and Sabah. Med. J. Malaysia..

[30] Yasin N.M., Abdul Hamid F.S., Hassan S., Mat Yusoff Y., Mohd Sahid E.N., Esa E. (2023). Characterization of New Alpha Zero (α0) Thalassaemia Deletion (--GB) among Malays in Malaysian Population. Diagnostics.

[31] Curinha A., Braz S.O., Pereira-Castro I., Cruz A., Moreira A. (2014). Implications of polyadenylation in health and disease. Nucleus.

[32] Clerici M., Faini M., Muckenfuss L.M., Aebersold R., Jinek M. (2018). Structural basis of AAUAAA polyadenylation signal recognition by the human CPSF complex. Nat. Struct. Mol. Biol..

[33] Molchanova T.P., Smetanina N.S., Huisman T.H. (1995). A second, elongated, alpha 2-globin mRNA is present in reticulocytes from normal persons and subjects with terminating codon or poly A mutations. Biochem. Biophys. Res. Commun..

