# Supplementary material for: The Clinical and Laboratory Profiles of a Deletional α2-Globin Gene Polyadenylation Signal Sequence (AATAAA > AATA--) [HBA2:c.*93_*94delAA]: The Malaysian Experience
Source: Diagnostics (Basel). 2025 May 20;15(10):1284. doi: 10.3390/diagnostics15101284 (PMC12110140; doi:10.3390/diagnostics15101284)
Supplement: Supplementary file 1 [file diagnostics-15-01284-s001.zip › diagnostics-3526734-supplementary.pdf]

a

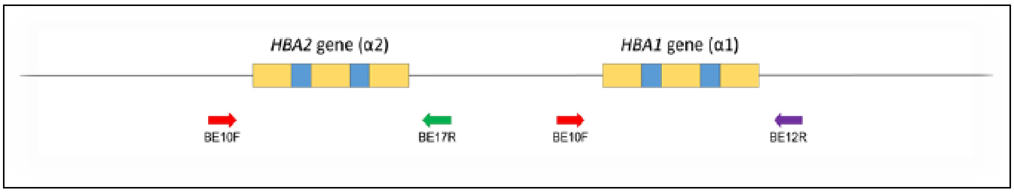

b

	Primer Name	Primer Sequence (5’ > 3’)
1.	BE10-F (forward primer for *HBA1* and *HBA2* genes)	TGG AGG GTG GAG ACG TCC TG
2.	BE17-R (reverse primer for *HBA2* gene)	CCA TTG TTG GCA CAT TCC GGG A
3.	BE12-R (reverse primer for *HBA1* gene)	CCA TGC CTG GCA CGT TTG CTG AGG
4.	AD Forward	CTTCGACCTGAGCCACGGCT

Supplementary figure S1: (a) The relative positions of the amplicon producing primers and (b) the primers sequence.
